# Physiological and Brain Activity After a Combined Cognitive Behavioral Treatment Plus Video Game Therapy for Emotional Regulation in Bulimia Nervosa: A Case Report

**DOI:** 10.2196/jmir.3243

**Published:** 2014-08-12

**Authors:** Ana Beatriz Fagundo, Esther Via, Isabel Sánchez, Susana Jiménez-Murcia, Laura Forcano, Carles Soriano-Mas, Cristina Giner-Bartolomé, Juan J Santamaría, Maher Ben-Moussa, Dimitri Konstantas, Tony Lam, Mikkel Lucas, Jeppe Nielsen, Peter Lems, Narcís Cardoner, Jose M Menchón, Rafael de la Torre, Fernando Fernandez-Aranda

**Affiliations:** ^1^University Hospital of Bellvitge-IDIBELLDepartment of PsychiatryBarcelonaSpain; ^2^Instituto Salud Carlos IIICIBER Fisiopatología Obesidad y Nutrición (CIBERObn)BarcelonaSpain; ^3^School of MedicineDepartment of Clinical SciencesUniversity of BarcelonaBarcelonaSpain; ^4^Instituto Salud Carlos IIICIBER Salud Mental (CIBERSam)BarcelonaSpain; ^5^University of GenevaGenevaSwitzerland; ^6^NetUnionLaussaneSwitzerland; ^7^Serious Game Interactive (SGI)CopenhagenDenmark; ^8^MobiHealth BVEnschedeNetherlands; ^9^IMIM (Hospital del Mar Medical Research Institute)Human Pharmacology and Clinical Neurosciences Research Group, Neuroscience Research ProgramBarcelonaSpain

**Keywords:** eating disorders, bulimia nervosa, emotional regulation, impulsivity, video game therapy, neuroimaging, fMRI

## Abstract

**Background:**

PlayMancer is a video game designed to increase emotional regulation and reduce general impulsive behaviors, by training to decrease arousal and improve decision-making and planning. We have previously demonstrated the usefulness of PlayMancer in reducing impulsivity and improving emotional regulation in bulimia nervosa (BN) patients. However, whether these improvements are actually translated into brain changes remains unclear.

**Objective:**

The aim of this case study was to report on a 28-year-old Spanish woman with BN, and to examine changes in physiological variables and brain activity after a combined treatment of video game therapy (VGT) and cognitive behavioral therapy (CBT).

**Methods:**

Ten VGT sessions were carried out on a weekly basis. Anxiety, physiological, and impulsivity measurements were recorded. The patient was scanned in a 1.5-T magnetic resonance scanner, prior to and after the 10-week VGT/CBT combined treatment, using two paradigms: (1) an emotional face-matching task, and (2) a multi-source interference task (MSIT).

**Results:**

Upon completing the treatment, a decrease in average heart rate was observed. The functional magnetic resonance imaging (fMRI) results indicated a post-treatment reduction in reaction time along with high accuracy. The patient engaged areas typically active in healthy controls, although the cluster extension of the active areas decreased after the combined treatment.

**Conclusions:**

These results suggest a global improvement in emotional regulation and impulsivity control after the VGT therapy in BN, demonstrated by both physiological and neural changes. These promising results suggest that a combined treatment of CBT and VGT might lead to functional cerebral changes that ultimately translate into better cognitive and emotional performances.

## Introduction

Conventional psychological therapies, such as cognitive behavioral therapy (CBT) have been successful in treating some central symptoms in eating disorders, such as binge/purging behaviors [1]. However, in bulimia nervosa (BN) patients, there are some dysfunctional features that still remain after treatment. Some of them, such as alterations in executive functioning (ie, impulsivity, planning, and decision making) and emotional deregulation (eg, self-control strategies or tolerance to frustration) are particularly difficult to modify and are associated with an adverse outcome [2].

PlayMancer is a video game designed to increase emotional regulation and reduce general impulsive behaviors, by training to decrease arousal and improve decision-making and planning [3,4]. As described in previous research [5], the final aim of video game therapy (VGT) is to achieve more efficient brain functioning with appropriate emotional and cognitive processing, which eventually translates into more suitable real-world behaviors. We have previously demonstrated the usefulness of PlayMancer in reducing impulsivity and enhancing emotional regulation in BN patients [6]. However, whether these behavioral improvements are also translated into changes in brain activity remains unclear.

This case report aimed at examining changes in physiological reactivity and brain activation as biomarkers of emotional regulation and impulsivity control in response to a combination of VGT and CBT treatments in a BN patient.

## Methods

### Case Report

#### Overview

The research procedures were explained in full to the patient and she gave written informed consent prior to enrollment in the study. The procedures were approved by the Ethical Committee of the University Hospital of Bellvitge. The patient was a 28-year-old woman seeking treatment for BN in our outpatient unit. BN was diagnosed according to the DSM-5 criteria [7]. The BN disorder started at the age of 22 (age of onset), after the patient had followed a hypocaloric diet. Pre-morbid overweight, a body mass index (BMI) of 28.7, and various psychosocial stressors were starter risk factors. The patient reported having started BN with 1-2 weekly bingeing and vomiting episodes. During approximately the last 6 months prior to the initiation of the treatments, the patient exhibited more than four weekly binge-eating episodes with compensatory fasting/restricting eating behaviors but without purging episodes, and continued to have extreme concerns about shape and weight. At the time of inclusion in the case study, her weight was 69.3 kg (height 166 cm, BMI 25.1).

#### Personal and Psychiatric Antecedents

The patient, the younger of two children, is currently living with her partner. Moreover, at the start of the treatment she did not present further additional psychiatric comorbidity or other familial psychiatric disorders, alcohol-drug misuse, regular tobacco consumption, or any other relevant difficulties for dealing with stress and negative emotions.

#### Psychometric Assessment and Physiological Measures

At the beginning, the patient was given the Eating Disorder Inventory [8], Symptom Checklist-90-Revised (SCL-90-R) [9], and Temperament and Character Inventory-Revised [10]. The psychometric assessment revealed a typical profile described frequently by BN patients (characterized by high body dissatisfaction, drive for thinness, bulimic episodes, social insecurity, anxiety, mild depressive symptoms, high harm avoidance, and low self-directedness). Comorbidity was assessed by means of the structured clinical interview for DSM-IV Axis I disorders (SCID-I/II). Additionally, weekly binge-eating and purging frequencies were recorded and monitored by means of a food diary throughout the duration of the therapy. Prior to and after VGT treatment, State-Trait Anxiety Inventory (STAI) [11] and Barratt Impulsiveness (BIS-11) [12] scales were administered. The physiological measures were analyzed by means of a sensor system via Bluetooth, linked to PlayMancer, including among others, autonomic measurements such as heart rate, pulse rate, and heart rate variability measures.

### Functional Magnetic Resonance Imaging Procedure

#### Paradigms

##### Emotional Face-Matching Task

To explore emotional activation and regulation, we used a modified version of the emotional face-matching task originally reported by Hariri, Bookheimer, and Mazziotta [13], which has also been reported elsewhere [14]. This task was proved to reliably activate the visual cortex, the amygdala, and the dorsolateral prefrontal cortex in healthy subjects. The contrasts of interest were fearful faces to shapes and happy faces to shapes. During each 5-second trial, the patient was presented with a target face (center top) and two probe faces (bottom left and right) and was instructed to match the probe expressing the same emotion to the target by pressing a button in either their left or right hand of a magnetic resonance imaging (MRI) compatible response device. The target face was either happy or fearful, and the probe faces included two out of three possible emotional faces (happy, fearful, and angry). As a sensorimotor control condition, the patient was presented with 5-second trials of ovals or circles in an analogous configuration and was instructed to match the shape of the probe to the target. A total of six 30-second blocks of faces (3 fearful, 3 happy) and six 30-second blocks of the control condition (shapes) were presented interleaved in a pseudo-randomized order. The contrasts of interest were fearful faces to shapes and happy faces to shapes.

##### Executive-Control Task

As an executive-control task, we employed the multi-source interference task (MSIT) [15], a task that reliably and robustly activates cingulo-frontal-parietal cognitive/attention network. Our contrast of interest was the incongruent condition to congruent condition. During each trial, the patient was asked to press one of three buttons in an MRI-compatible response device to identify the unique digit (1, 2, or 3) that was not repeated in a string of three digits. Each digit was mapped to index (1), middle (2), and ring (3) fingers of the right hand and subjects should respond to the identity (not the position) of the unique digit in the string. There were a total of 48 congruent trials (ie, 133, 121, 223) and 48 incongruent trials (ie, 212, 311, 322), distributed in a total of eight blocks of 12 trials each, separated with nine fixation crosses of 15-second duration. In the incongruent situation, the unique digit’s spatial position in the string was conflicted with the corresponding identity of the unique digit. Our contrast of interest was the incongruent condition to congruent condition.

#### Acquisition, Processing, and Analyses of the Images

The patient was scanned twice in a 1.5-T Signa Excite system (General Electric) Magnetic Resonance (MR) scanner, prior to and after the 10-week VGT/CBT combined treatment. The MR was equipped with an 8-channel phased-array head coil and single-shot echoplanar imaging software was used. The functional sequence consisted of gradient recalled acquisition in the steady state (repetition time=2000 ms, echo time=50 ms and pulse angle, 90º) in a 24 cm field of view, with a 64 x 64 pixel matrix, and a slice thickness of 4 mm (inter-slice gap, 1.5 mm).

A total of 22 interleaved sections, parallel to the anterior-posterior commissure line, were acquired to generate 207 (MSIT task) and 195 (emotional face-matching task) whole-brain volumes. Visual stimuli were presented using MRI-compatible goggles (VisuaStim Digital System, Resonance Technology Inc, Northridge, CA, USA), while behavioral responses were recorded by means of an MRI-compatible response grip (NordicNeuroLab Inc, Bergen, Norway). Imaging data were processed on a Macintosh platform running Matlab version 7 (The MathWorks Inc) and statistical parametric mapping software version 8 (SPM8). Time series of the pre and post acquired images were initially realigned to the mean image by using a 6-parameter (rigid body) spatial transformation, normalized to the standard eco-planar imaging template in SPM, resliced in Montreal Neurological Institute (MNI) space and smoothed using a Gaussian filter (full width at half maximum=8 mm). Realigned, normalized, and smoothed images were carried to a first level of analysis for the contrasts of interest in each task. Results were thresholded at a positive false discovery rate<0.05 corrected. Finally, the pre and post VGT/CBT combined treatment were overlapped in a T1 MNI template in MRIcron software (Rorden and Brett, 2000) for visual comparison and presentation of the results.

### Treatment

**Figure 1:**
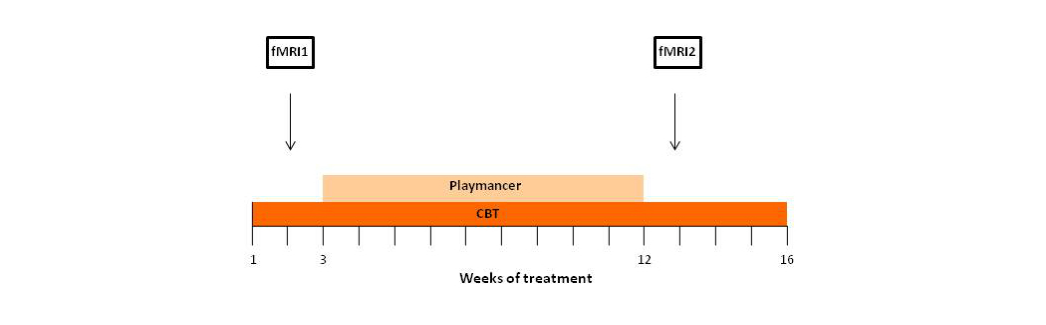
Schematic diagram of treatments and procedures.

As described previously [6], a combined therapy was used in this case study (16 weekly outpatient cognitive-behavioral group sessions plus 10 weekly sessions of VGT) (Figure 1). A detailed description of the main goal, techniques, and structure of the CBT group therapy have been described previously [1], as well as the VGT approach used [3,4].

**Figure 2:**
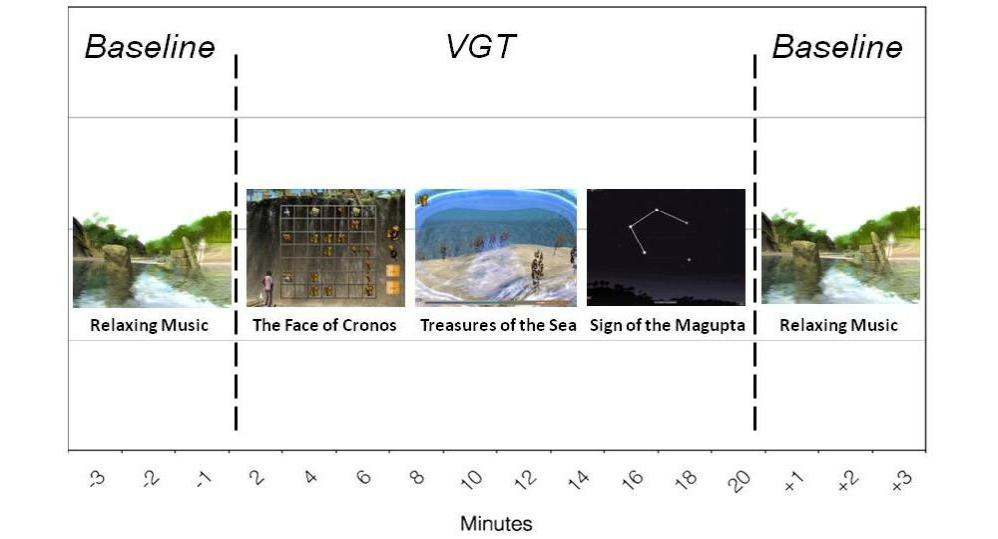
Example of PlayMancer session: relaxing phases and mini-games.

The performance in each VGT session was collected during 20 minutes. Three minutes of relaxing music were played before and after the VGT session. The video game consisted of three mini-games: (1) The Face of Cronos, where the player has to climb up a cliff in which obstacles appear depending on the arousal of the player (based on biofeedback); this mini-game trains planning and decision making, (2) Treasures of the Sea, which is a virtual swimming game in which the player has to collect different objects and fishes while conserving their oxygen supply; this trains visuospatial abilities, visual working memory, and decision making, and high arousal makes the task more difficult, and (3) Sign of the Magupta, which is a relaxation game in which the player connects a constellation of stars through breathing control; slow deep breathing allows the connections between stars to form [6] (see Figure 2 and Multimedia Appendix 1).

## Results

### Eating Symptomatology, Anxiety, and Impulsiveness

During the combined treatment, the rate of binge eating and the consequent compensatory behaviors started to decline after the fourth session, whereas abstinence of bingeing occurred after the sixth session. At the 6- and 12-month follow-ups, the patient was still free of binge eating symptoms. Regarding secondary outcome measures (namely anxiety and impulsivity), the patient had reduced impulsivity (measured by means of BIS-11, pre mean score 38 and post mean score 29, with the Spanish population mean score of 32.5, Oquendo et al, 2001) and state of anxiety (measured by means of STAI, pre mean 38, post mean 17) after VGT intervention, and it was maintained after follow-up.

### Evolution of Physiological Variables With the Video Game Therapy

**Figure 3:**
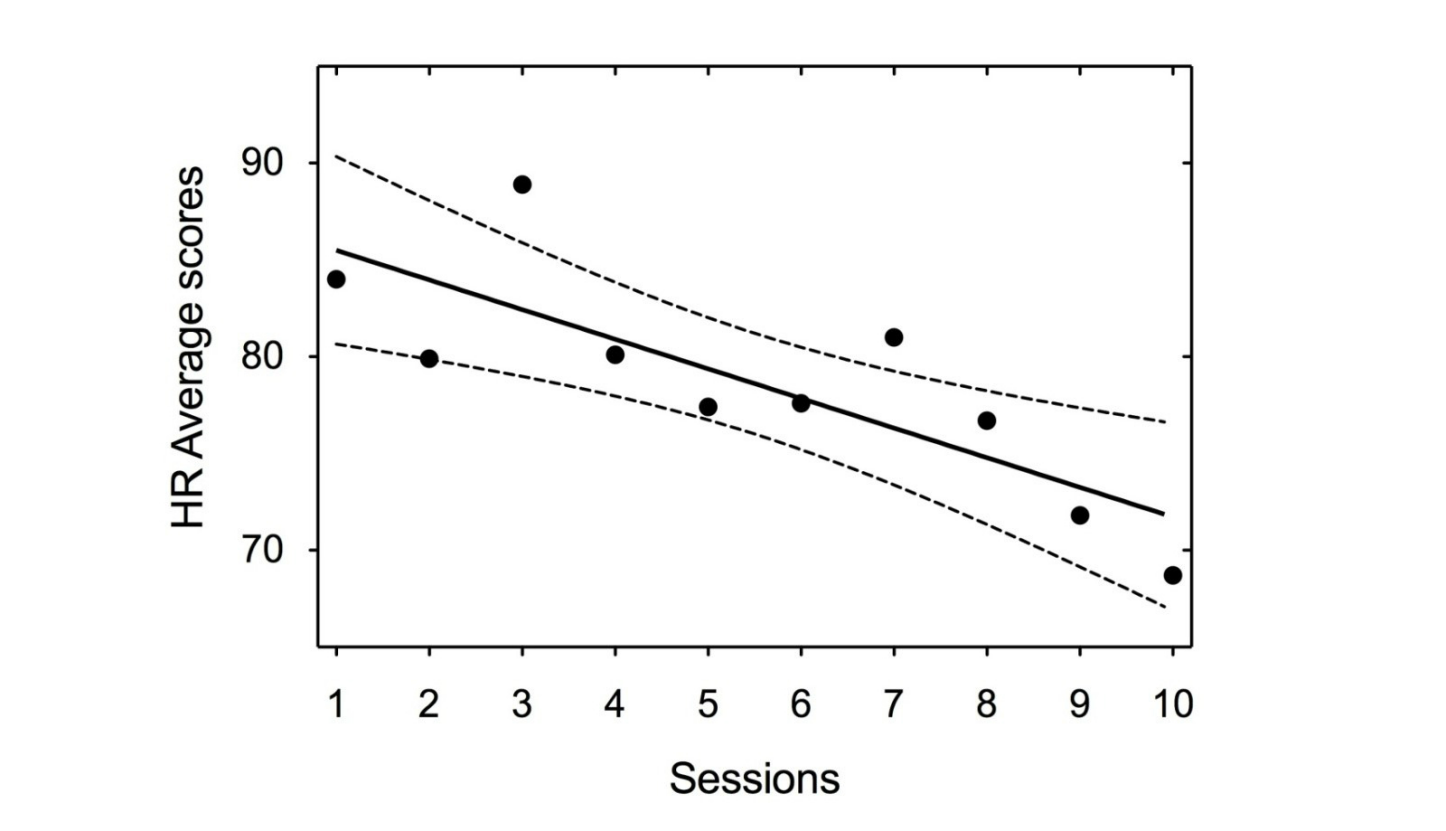
Average Heart Rate (HR) scores over the 10 therapy sessions.

A trend was found for the weekly average heart rate (HR), which signified scores tended to decrease with the game sessions (Figure 3).

### Internal Outcome Measure of the Video Game Therapy

**Figure 4:**
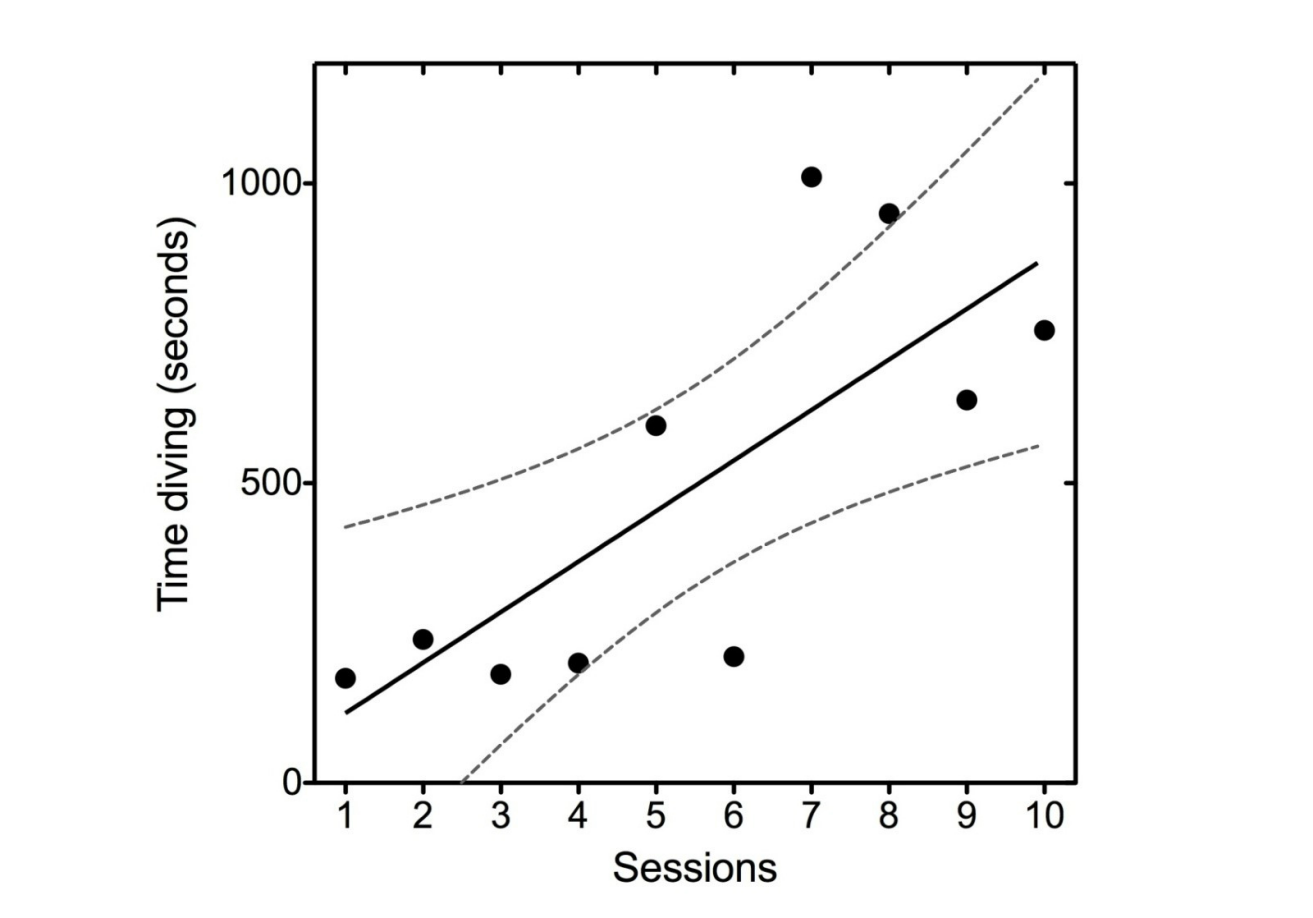
Video game internal secondary outcome measure (average time diving in seconds over the 10 therapy sessions).

One of the internal VGT measures was the total time spent in a specific task (diving task), which is the absolute diving time (in seconds) the subject plays without being interrupted (due to lack of stress management or emotional regulation capacity, oxygen ran out, and the diving session was interrupted). A positive trend was found for the weekly average total diving time, which means scores tended to increase with the sessions of the game (Figure 4).

### Pre-Post Changes in Brain Activity

Behavioral measures indicated that reaction time decreased in the post condition in both the face-matching task and the MSIT task (see Table 1). The patient had high global accuracy in both tasks (face-matching tasks: pre: 97.22%, post: 98.61%; MSIT: pre: 100%, post: 97.92%).

**Figure 5:**
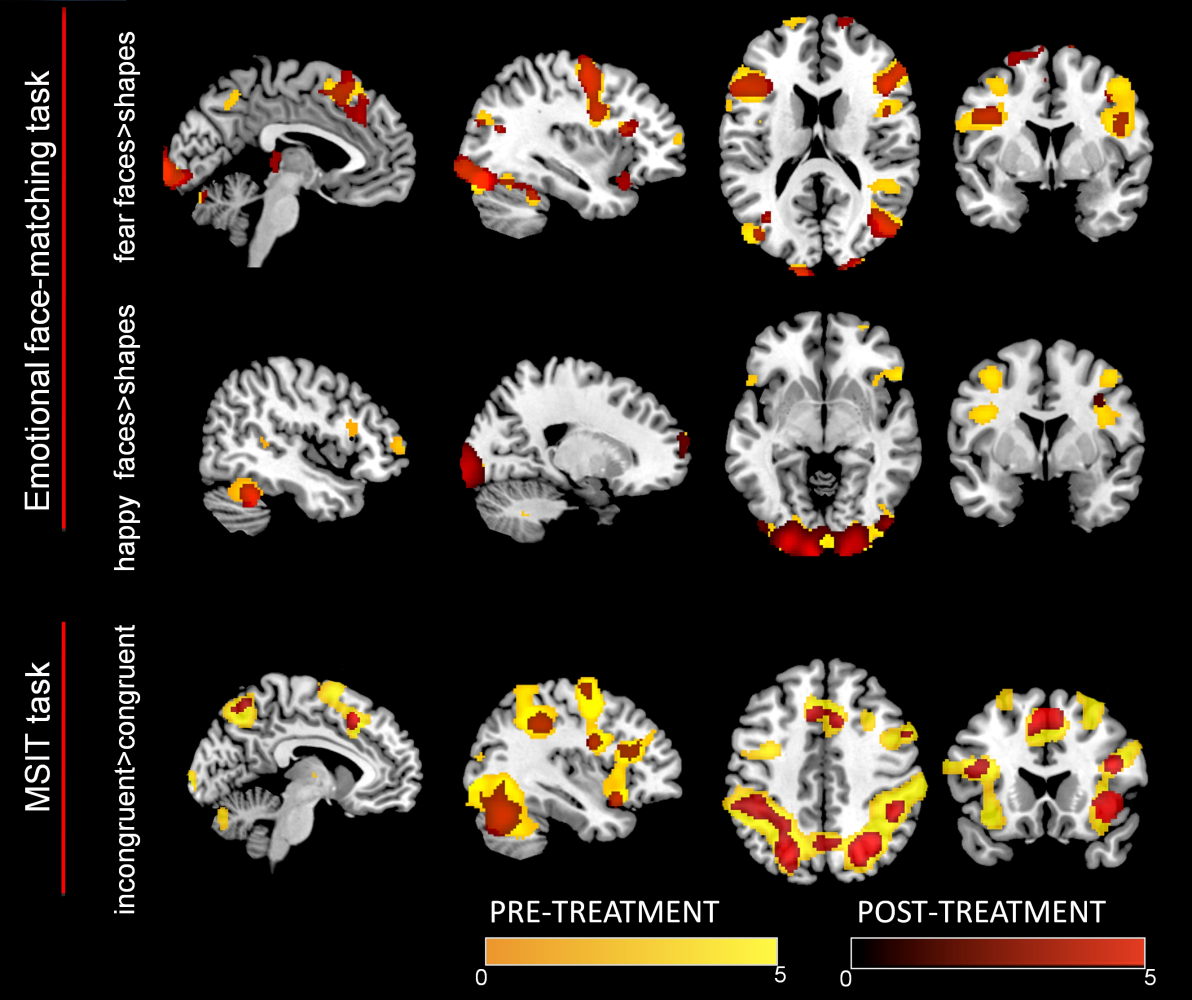
Pre-treatment and post-treatment activations during the emotional face-matching task (happy faces vs shapes and fearful faces vs shapes) and the multi-source interference (MSIT) task (incongruent vs congruent condition).

The face-matching task engaged areas typically active during this task in healthy controls, such as fusiform gyrus, visual cortex, precentral and dorsolateral prefrontal cortices (see Figure 5). Pre and post conditions were partially overlapping, although, globally, the cluster extension of the active areas decreased after combined treatment. A small cluster in the left amygdala (2 voxels, see Figure 5) was also active during the fearful faces matching, even though only in the pre treatment condition (matching happy faces during the pre condition and fearful and happy faces during the post condition did not activate the amygdala, even when lowering the threshold to an uncorrected *P*<.001). Other differences between pre and post conditions were located in the bilateral dorsolateral prefrontal cortex during the matching of fearful faces and in the bilateral frontopolar-anterior insula /bilteral dorsolateral prefrontal cortex during the matching of happy faces.

**Figure 6:**
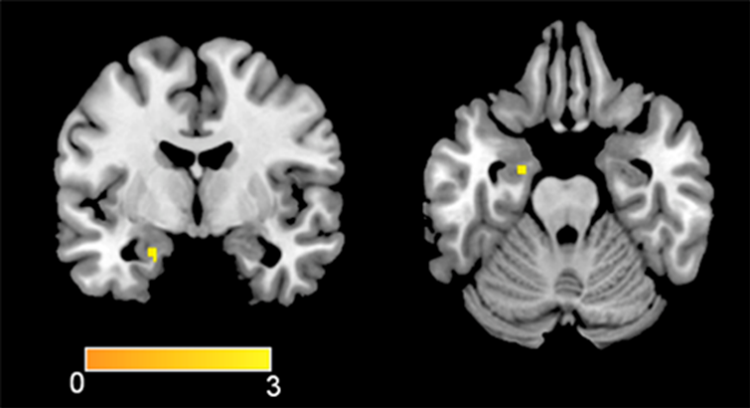
Activations in the bilateral anterior insula after the treatment.

With regard to the MSIT task, the incongruent versus congruent conditions showed activations in the medial prefrontal-dorsal anterior cingulate and superior parietal cortex, for both pre and post assessments (see Figure 5). Although these areas are typically activated in healthy controls while carrying out the task, again at post assessment the patient engaged a smaller cluster extend of voxels. Additional activations were found in the bilateral anterior insula, which were also found to decrease after the combined treatment (Figure 6).

**Table 1:** Pre-post changes in brain activity (fMRI^a^): behavioral measures.

Paradigm		Pre, mean (SD)^b^	Post, mean (SD)^b^
**Face-matching task**			
	Fearful faces trials	1.44 (0.07)	1.12 (0.04)
	Happy faces trials	1.00 (0.05)	0.91 (0.03)
	Shapes trials	0.72 (0.03)	0.68 (0.08)
**Multi source interference task (MSIT)**			
	Congruent trials	0.542 (0.009)	0.478 (0.005)
	Incongruent trials	1.104 (0.063)	0.917 (0.082)

^a^fMRI: functional magnetic resonance imaging.

^b^Results are presented in seconds.

## Discussion

### Principal Findings

This case study set out to examine the neural and physiological changes associated with a combined therapy (CBT plus video game therapy), as a tool to enhance emotional regulation and impulsivity control in a BN patient. In this reported case, the results suggest that specific training to decrease arousal and increase impulsivity control [6] may improve emotional regulation, and induce changes in physiological variables (eg, HR) and in the neural circuits related to emotional and executive processing. Although emotional regulation has been previously studied in BN [6], and new technologies have been previously used in psychology [16,17], this is, to the best of our knowledge, the first time that the neural and physiological changes associated with a combination of VGT and CBT have been described in a BN patient.

According to our results, self-regulation training incorporating physiological and emotional feedback might improve the emotional regulation capacity. Specifically, the reduction of physiological markers (eg, HR) after the VGT may suggest higher emotional control and is in agreement with those studies showing that a dysfunctional control over emotions is associated with increased heart rate, heart rhythm dysregulation, and autonomic imbalance [18,19]. In this regard, recent evidence indicates that decreases in physiological variables, such as HR, are connected not only to a higher self-regulation of emotions, but also with specific improvements in executive functions (eg, cognitive flexibility and control over impulsive behaviors) [20], which is also in line with the neuroimaging results in this case report.

In this sense, after the combined VGT/CBT treatment, the patient was able to display a better behavioral performance on both the video game tasks and the functional magnetic resonance imaging (fMRI) paradigms. Specifically, an enhancement of absolute time playing the game and lower reaction times with high global accuracy in the fMRI paradigm were observed. As explained above, PlayMancer is a video game specifically designed for training emotional regulation, but can also be used for training planning, inhibition response, decision making, and working memory capacities [6]. Thus, these findings suggest that in this BN case, the combination of CBT with regular cognitive training improves cognitive functions and produces changes in the neural substrates associated.

Specifically, the neural patron displayed by the patient while performing the fMRI paradigms was similar to the one found in healthy controls, for both the impulsive control paradigm (MSIT) [15] and the emotional paradigm [14], but was modified after the combined treatment. Executive and attention networks were active during emotion processing and cognitive interference tasks. However, on the one hand, it is suggested that brain activations were more efficient after the combined treatment, given that an improvement of behavioral results was achieved with a lesser extent of cluster activations. The amygdala also showed some differences between the pre and post conditions of the fearful face-matching trials. Although the cluster extent of the amygdalar activation was small, it may also suggest some differences in emotional regulation between the pre and post treatment conditions.

On the other hand, the activation of the anterior insula during cognitive conflict (incongruent condition versus congruent condition) may be consistent with the idea that additional brain resources were needed to perform the tasks. Even though the anterior insula is not generally activated by the MSIT [15], it has been found to be active during performance monitoring and is modulated by error awareness [15]. Additionally, the anterior insula is suggested to be a key dysfunctional structure in the pathophysiology of eating disorders [21].

In summary, these promising results suggest that, in this case report, a combined treatment of CBT and VGT might lead to functional cerebral changes that might eventually translate into better cognitive and emotional performances. This report emphasizes the importance of researching new treatments for enhancing emotional regulation and impulsivity control in BN patients.

### Strengths and Limitations

This case study also has several important strengths, primarily the novelty of the therapeutic approach. VGT, as applied in the present case study, might be a practical tool for the treatment of cognitive and emotional alterations in BN.

However, the results of this case study should be interpreted within the context of some limitations. The most important one is that it is a single case report study, although a longitudinal design was employed and pre-post measures of the patient were considered. However, brain activity pre-post was not statistically compared, thus future studies evaluating series of cases-controls should be conducted in order to confirm these findings. Additionally, although the repetition of the task may be contributing to the improvement in brain and behavioral efficiency, this effect could not be differentiated here and would need to be further tested in a case-control study.

### Conclusions

Though this report exemplifies a novel treatment for cognitive and emotional rehabilitation in BN patients (ie, video game therapy), more studies need to be carried out and future neuropsychological and neuroimaging studies should focus on the executive and emotional profile of these patients, in order to shed more light on these multifaceted constructs.

## Multimedia Appendix 1

Video game PlayMancer.

## Abbreviations

BIS-11: Barratt Impulsiveness Scale
BMI: body mass index
BN: bulimia nervosa
CBT: cognitive behavioral therapy
fMRI: functional magnetic resonance imaging
HR: heart rate
MRI: magnetic resonance imaging
MSIT: multi-source interference task
STAI: State-Trait Anxiety Inventory
VGT: video game therapy
